# Past, Present and Future: A Historical Analysis and Reflection of 100 Years of Nursing Leadership

**DOI:** 10.1111/nin.70152

**Published:** 2026-08-02

**Authors:** Sherrie Bolton, Amy‐Louise Byrne, Lisa Wirihana, Justine Connor, Ellie Cutmore

**Affiliations:** ^1^ School of Nursing, Midwifery and Social Sciences CQUniversity Rockhampton Queensland Australia; ^2^ School of Nursing, Midwifery and Social Sciences CQUniversity Sydney New South Wales Australia; ^3^ School of Nursing, Midwifery and Social Sciences CQUniversity Brisbane Queensland Australia

**Keywords:** historical analysis, leadership, leadership styles, leadership theories, nursing history, nursing leadership

## Abstract

This paper critically examines the evolution of nursing leadership over the past century, exploring how changing historical, social and professional contexts have influenced styles and their ongoing relevance to contemporary nursing practice. Using a historical analysis guided by Lewenson and Hermann's framework, primary sources, including archival materials and foundational texts, were examined alongside secondary scholarly literature and historical commentaries. Leadership approaches were reviewed by decade and mapped against key nursing theories, healthcare developments and broader social movements to construct a timeline of leadership discourse. Findings demonstrate a progression from predominantly hierarchical, autocratic and bureaucratic leadership models characteristic of the early and mid‐20th century, particularly during wartime and post‐war periods, toward more relational, collaborative and adaptive approaches. Transformational leadership emerged alongside nursing professionalisation during the 1980s, while servant and authentic leadership gained prominence in response to growing emphasis on equity, inclusion and person‐centred care in the 2010s. More recently, adaptive, emotionally intelligent and ambidextrous leadership styles have become increasingly important for addressing healthcare complexity, workforce resilience, innovation and change. The analysis highlights nursing leadership as a dynamic, context‐dependent and deeply relational construct. Understanding its evolution can support nurse leaders, educators and other healthcare providers to critically reflect on practice, foster inclusive and resilient teams and apply evidence‐informed leadership strategies that promote sustainable, patient‐centred care in rapidly changing healthcare environments.

## 1 Introduction

Leadership in nursing is a critical determinant of healthcare delivery, influencing patient outcomes, staff satisfaction and organisational performance. Effective nurse leaders navigate complex clinical environments, foster collaboration and promote evidence‐based nursing practice, leading to the quality and outcomes for patients. Leadership is part of a complex fabric, further influenced by factors including autonomy, competency, relatedness, professional relationships, support and leadership style (Alsadaan et al. 2023; Al‐Rjoub et al. 2024). These coalesce to ensure that care is fit for purpose, safe and effective, highlighting the importance of effective and supportive leadership.

Leadership in nursing has never been static. It has evolved in response to the shifting tides of history, social norms and healthcare priorities. From the rigid hierarchies of the early 20th century, shaped by wartime urgency and military influence, to the relational and adaptive models of today, nursing leadership reflects the broader sociocultural context in which it operates (Gebbie 2009). Nursing leadership has evolved alongside changes in nursing education. Early practice relied on hospital‐based apprenticeship models, which were task‐oriented and subordinate to medical authority, reinforcing hierarchical structures and autocratic norms during wartime and post‐war periods (Clark 1995; Gebbie 2009; Kanaris 2023). The mid‐20th century saw professionalisation through university programs, fostering autonomy and critical thinking (Stievano et al. 2019). This shift aligned with transformational leadership, as nurse leaders were expected to inspire innovation and advocate for professional identity (Bass 1985; Gabel 2013). Later, globalisation and technological advances expanded curricula to include research, ethics and leadership theory, embedding participative and shared governance models (Scott and Caress 2005). Today, education emphasises person‐centred care, cultural safety and resilience, supporting leadership approaches such as authentic and servant leadership (McCormack and McCance 2010; van Dierendonck 2011). Understanding this historical trajectory is essential because leadership styles are not merely managerial choices; they are deeply embedded in the cultural and institutional legacies of the profession, which makes leadership today, complex and complicated requiring flexibility.

Persistent global challenges, related to nursing workforce shortages and disengaged professional culture, highlighting the critical need for supportive, sustainable work environments (Speroni 2024). The pivotal role of leadership in cultivating and maintaining healthy nursing workplaces is well documented; however, styles vary often reflecting the historical roots of nursing and social norms of the era (Cummings et al. 2018). Leadership in nursing is viewed as a relational, context‑dependent practice shaped by historical, professional and sociocultural forces, rather than a fixed managerial role. Different eras and settings demanded different leadership approaches, expressed through influence, values and everyday interactions across all levels of practice. While organisational structures and management roles appear in the discussion, managerial functions are not examined on their own, they are considered only where they intersect with leadership and reflect broader leadership discourses in nursing.

This paper explores the diverse landscape of leadership styles and their evolution over the years, paying careful attention to where the social epochs within leadership movements have existed. The intent of the article is to explore nursing leadership, allowing space for critical reflection on leadership styles and practice. The paper adopts a critical, relational view of leadership, understanding it as socially constructed, historically situated and shaped by power relations within healthcare systems (Kok et al. 2023). Drawing on critical nursing scholarship, leadership is examined not as a neutral or managerial function but as a practice embedded in sociopolitical, gendered and institutional contexts. This framing guides the historical analysis, which considers not only shifts in leadership styles but also the conditions that produced them. Leadership approaches are interpreted as responses to specific historical pressures, including war, professional regulation, equity movements and major healthcare reforms.

This analysis is situated within Western Anglophone healthcare contexts, such as the United States, United Kingdom, Canada, Australia and New Zealand, where much of the nursing leadership literature and professional documentation has historically emerged (see Table 1). Accordingly, the developments discussed should not be assumed to apply universally across all nursing traditions. While the paper traces a broad historical chronology, it does not presume a single global trajectory of leadership. Instead, leadership change is understood as contingent, negotiated and sometimes resistant, shaped by power relations within healthcare systems rather than progressing linearly toward ‘better’ models (Uhl‐Bien et al. 2020). This perspective informs the analytical connections drawn across periods.

**Table 1:** Mapping of leadership theories and social/cultural movements by decade.

Decade(s)	Leadership styles and theories	Leadership features	Nursing theories	Social and cultural interventions
1920–1950	Autocratic	Nursing was highly hierarchical, with strict physician–nurse dynamics	Transactional	Military and religious roots of hierarchy
	Trait theory	Command‐and‐control, task delegation, limited autonomy	Apprenticeship style	Efficiency in wartime and hospital settings, but low staff empowerment
	Transactional			
	Leadership theory			
1960–1970	Bureaucratic	Emphasis on rules, policies and structured supervision	Watson theory of caring	Civil rights
	Role‐based leadership	Expansion of nursing education and formal roles	Bureaucratic leadership theory	Second‐wave of feminism
		Standardisation of care, but limited innovation or collaboration		Countercultural and unrest post‐wars
1980–1990	Transformational leadership	Shift toward professionalisation and nursing theory development	Benner Novice to Expert	Nursing moved from job‐based to university training
		Vision‐driven, motivational, focused on change and innovation	Jean Watson's theory of human caring	Globalisation and digitisation—home computers and entry tech
		Increased job satisfaction, better interdisciplinary communication	Dorothea Orem's self care deficit nursing theory	Internet revolution
				Acquired Immuno‐Deficiency Syndrome (AIDS) as a global epidemic
2000	Participative and shared governance models	Collaboration, shared decision‐making, team empowerment	Initial introduction of person‐centred nursing theory	Terrorism and war
	Laissez‐faire leadership	Increased job satisfaction, better interdisciplinary communication	Tim Porter O'Gradys shared governance theory	Social media
				Financial crisis and economic disparity gaps
				Quality and safety movement increased patient participation and introduction of person‐centred models Close the gap
2010	Authentic and servant leadership	Focus on emotional intelligence, ethics and staff well‐being	Relational leadership theory Further development of person‐centred nursing theory	Smart phone and digital saturation
		Self‐awareness, transparency, service to others	Self determination theory	Equity focus—LGBTIQ, Indigenous and minority rights
		Reduced burnout, improved trust and psychological safety	Resilience in nursing, burnout	Black Lives Matter and #Metoo
2020	Adaptive, ambidextrous and emotionally intelligent leadership	Flexibility, empathy, innovation and resilience	Complexity leadership theory	COVID‐19, digital transformation, generational shifts
		Enhanced patient safety, staff retention and organisational agility	Ambidextrous leadership theory	Increased political violence
				Rapid advancement of artificial intelligence

## 2 Background—What Is ‘Leadership’?

A leadership style refers to the characteristic approach or method a leader employs to guide, influence and manage individuals or teams toward achieving goals (Reinhardt et al. 2022). It encompasses the leader's behaviour, decision‐making patterns, communication strategies and motivational techniques (Reinhardt et al. 2022). In nursing, leadership style directly impacts patient outcomes, staff satisfaction and organisational culture (Reinhardt et al. 2022). Importantly, leaders are not necessarily managers, and through the years the definition of leadership has changed; however, it is commonly understood as guiding people toward a shared goal (Metcalf and Benn 2013). In this paper, a nurse leader is not defined solely by formal position or title. Leadership is understood as a practice enacted through influence, decision‐making, advocacy and professional authority, which may occur within or beyond designated managerial roles (Reinhardt et al. 2022). Accordingly, nurse leaders in this analysis may include individuals in formal positions (such as nurse managers, educators and executives), as well as those who exercised leadership through professional advocacy, education, regulation, military service or workforce organisation.

According to Val and Kemp (2012), three predominant styles form a spectrum of leadership with one end of the spectrum being autocratic, followed by democratic and progressing to the other end being laissez‐faire. Autocratic leadership adopts an authoritarian approach, where the leader makes decisions for the team (Val and Kemp 2012). Democratic leadership is participative, where the leader takes a relaxed but in‐control approach, consulting the team before making a decision. Laissez‐faire leadership reflects a hands‐off approach, where participants resolve their problems with minimal guidance (Val and Kemp 2012). Effective nurse leaders often blend styles to meet the demands of complex environments (Reinhardt et al. 2022).

Leadership itself is a complex relationship between leaders and followers, being shaped by external and internal influences, the authority to lead, cultural norms, social positioning and other factors (Coleman et al. 2024). A leader (and their leadership) cannot exist in isolation, and as such it is a composite of power and relationships. Leaders must navigate complex interpersonal dynamics, ethical considerations and ever‐changing environments with this complexity shaping how leadership is seen today, and the way nurse leaders respond to dynamic demands (Van Vugt et al. 2008). Leadership is not a one‐size‐fits‐all concept, as in the dynamic, evolving environments of healthcare, the ability to adapt leadership approaches to suit specific contexts is essential. Many contemporary societal and nursing theories have emerged to inform leadership in various contexts, for example, transactional leadership theory, Watson's theory of human caring and person‐centred nursing theory (Antonakis and House 2014; Gunawan et al. 2022; McCance and McCormack 2025). These theories have evolved with the profession, providing foundational perspectives on care delivery and what it means to be a nursing leader, highlighting the changing landscape of leadership in the dynamic environment within which it exists (Antonakis and House 2014; Gunawan et al. 2022; McCance and McCormack 2025).

The purpose of this paper is to critically examine nursing leadership styles through a reflective lens, exploring different perspectives on leadership, including styles and theories and their relevance to contemporary nursing practice. Rather than advocating for a single approach, we aim to explore the types of nursing leadership in context, with the view of igniting reflection. Importantly, this article does not seek to develop new leadership theories or prescribe a singular model for future practice. It is not empirical in nature. Rather, its purpose is to provide a historical and reflective analysis of nursing leadership, tracing its evolution across social and professional contexts and inviting readers to critically consider how these legacies shape contemporary leadership approaches now and into the future. We recognise the substantial body of work on nursing leadership, and the wide range of nursing theories which inform leadership today.

## 3 Methodology

This study uses approaches from nursing historiography to conduct an interpretive historical analysis of leadership development. Lewenson and Herrmann's *Capturing Nursing History* (2008) informs the approach to contextual interpretation, source critique and synthesis, rather than serving as a strict methodological framework. The study draws on secondary analyses of historical and leadership literature, supplemented by published primary sources such as foundational leadership texts, professional reports and policy documents. These materials were examined using established historical interpretation techniques, including attention to context, authorship, purpose and audience.

Situated within history as a disciplinary field, this paper adopts a historical analysis to examine the evolution of nursing leadership over time, as described by Lewenson and Herrmann (2008), to explore the evolution of nursing leadership styles over the past century. Historical analysis systematically examines past events, theories and practices to understand how leadership models have emerged, transformed and influenced nursing practice across different eras (Lewenson and Herrmann 2008). This approach allows for a contextualised understanding of nursing leadership, tracing its evolution in response to sociocultural shifts, healthcare reforms and changes in professional identity. Theoretical perspectives were used as interpretive tools rather than standalone frameworks. Leadership theories and critical concepts were read alongside historical events to show how leadership discourses aligned with or challenged prevailing social and professional norms. Social and cultural influences were identified through review of nursing history literature and crosschecked against leadership and policy developments. Movements shown to shape nursing practice, identity or health system structures were included. The analysis draws primarily on Western leadership literature selected for its influence within those healthcare contexts.

### 3.1 Aim

This article aims to map and critically explore the history and evolution of leadership in nursing, with the view of understanding how the social context of the profession has shaped leadership styles. The secondary aim is to drive discussion and reflection about the changes in leadership discourses within contemporary and future nursing.

### 3.2 Methods

The research approach was structured around key periods in nursing history across the last 100 years, drawing on both primary and secondary sources. Sources were purposively selected for their relevance to leadership discourse and historical influence, with reference chaining used to identify seminal texts. Primary sources included foundational leadership writings, professional reports and policy documents, and secondary sources comprised historical nursing scholarship and leadership analyses. Limited early‑20th‑century archives meant interpretations of that period relied mainly on secondary accounts, with primary materials increasing from the mid‑century onward. Leadership styles are therefore discussed as features of historical analysis rather than empirically verified behaviours. The analysis interprets broad patterns in leadership discourse, not individual leaders' practices or universal trends across decades.

This method enabled a nuanced understanding of how leadership styles both shaped and were shaped by sociocultural shifts, healthcare reforms and cultural expectations of an era. It also facilitated exploration of their influence on nursing workforce dynamics, professional autonomy and leadership identity. By tracing the historical trajectory of leadership in nursing, this paper illuminates the contextual factors that have informed leadership practices and provokes critical reflection on their implications for contemporary and future nursing leadership. While events such as wartime mobilisation are discussed in relation to leadership arrangements, this analysis does not attribute any leadership style to a single causal factor. Fully accounting for the political, economic, gendered, racial, educational and professional forces shaping leadership in each era lies beyond the scope of this paper. Factors such as medical dominance, gendered labour relations, racialised exclusions, shifts in nurse education and changes in regulation all influenced leadership in uneven and contested ways (Stievano et al. 2019; Gebbie 2009). Rather than disentangling these influences exhaustively, the paper focuses on how leadership styles were represented within historical analysis. Social and cultural interventions were identified through iterative reading of nursing history literature and cross‐referenced against leadership and policy developments occurring within the same periods. Those movements repeatedly identified as influencing nursing practice, professional identity or health system structures were included.

### 3.3 Process

An initial search of the literature identified common leadership styles, which were leveraged to explore and expand additional leadership styles. Primary and secondary sources were searched and reviewed by decade, providing a timeline of nursing leadership and facilitating discussion on its evolution. Major nursing theories were mapped by decade, then expanded into a historically and socially relevant discussion. Under the historical analysis, the literature review was not systematic, where choices are justified and recorded against a pre‐defined criterion. Instead, an iterative process of exploring and mapping, within the historical analysis lens, guided development of a comprehensive timeline of nursing leadership. The intention was not to be exhaustive with leadership style, but to trace major leadership discourses and associated social movements across the nursing profession (Lewenson and Herrmann 2008). Consistent with historical analysis by Lewenson and Herrmann (2008), the literature review reflects dominant leadership discourses within healthcare systems rather than a comprehensive cross‐national comparison. The construction of the leadership timeline (Table 1) was informed through iterative comparison of leadership discourse, nursing theory and sociopolitical events identified within the literature. Leadership styles and theoretical developments were included in the timeline when they were consistently referenced across multiple sources as influential within nursing practice or professional discourse during that period.

## 4 Findings—Nursing Leadership in Time

Mapping allowed for a contextualised exploration of the evolution and history of nursing leadership. As per Table 1, the leadership styles, features, theories and social influences coalesced to form new understandings of nursing leadership. The mapping shows that leadership developments often followed broader societal changes, indicating a temporal lag between sociopolitical movements and shifts in nursing leadership discourse. This reflects the historically contingent and institutionally mediated nature of leadership development. Decade groupings in this analysis represent recurring patterns in leadership discourse rather than homogeneity within periods. Periods are grouped when leadership approaches share common structural logics, organisational arrangements and professional expectations in dominant literature and policy narratives. For example, despite significant disruption from the 1920s–1950s, leadership discourse consistently emphasised hierarchy, role stratification, discipline and task orientation (Clark 1995; Strahan 1997), justifying their analytic grouping. Such grouping highlights continuity in leadership norms without implying uniformity or stasis.

### 4.1 The 1920s to 1950s—Structure and Order

#### 4.1.1 Autocratic Leadership

Historically, healthcare organisations were built on a rigid structure of authority and power (Harding 2005). The outbreak of the World Wars resulted in an unprecedented demand for nurses, many of whom were drawn into military service (Strahan 1997). Autocratic leadership, first defined by Kurt Lewin in 1939 within his Leadership Theory Framework, is characterised by a directive and authoritarian approach in which leaders maintain substantial control over decision‐making and enforce strict compliance (Al‐Thawabiya et al. 2023). Autocratic leaders often exhibit traits such as time urgency, authoritarian tendencies, limited consideration for colleagues' needs and rejection of shared decision‐making (Briker et al. 2021). This is articulated by Stievano et al. (2019, 26).

The major societal turns, such as wars, led social changes that encouraged nursing professionalism. For example, after World War I, the social advancements of women were tangible in many countries, influencing in different ways nursing professionalization.

The strong military influence on nursing during this period shaped organisational structures and normalised hierarchical leadership arrangements. In this analysis, these arrangements are interpreted as aligning with features later described as autocratic, without assuming that individual nurse leaders uniformly enacted autocratic styles. Although the prominence of autocratic leadership within the military between 1920s and 1950s influenced nursing leadership, this approach was arguably already embedded in nursing. Florence Nightingale's early model of nursing leadership reflected an autocratic approach, characterised by hierarchical systems and authoritative control (Clark 1995). The military influence during this era likely strengthened, rather than introduced, this existing leadership paradigm. Florence Nightingale's early nurse‑training model established rigid hierarchies shaped by class, gender and social order within British hospitals (Clark 1995; Nelson 2003). Middle‑class women occupied supervisory and instructional roles, while working‑class women were largely excluded from formal leadership opportunities (Clark 1995; Nelson 2003).

During this time, gendered assumptions influenced preferences for leadership styles. Male leaders were associated with autocratic methods, reflecting perceptions of men as hierarchical, less collaborative and less attuned to team dynamics (Vinnicombe 1988). At the time, and still today, leadership was associated with masculinity, aligning with early‐20th‐century social norms when women had fewer rights and opportunities than men (Elliott et al. 2024). Despite nursing being a female‐dominated profession, its military association during this era likely reinforced adoption of traditional, masculine leadership styles (Elliott et al. 2024). Despite nursing's female dominance, its wartime ties to military and state institutions introduced leadership norms centred on hierarchy, discipline and centralised authority (Strahan 1997; Nelson 2003). Although often characterised as masculine, wartime nursing also expanded many nurses' autonomy, responsibility and public visibility, complicating any singular view of these norms as uniformly restrictive (Strahan 1997; Nelson 2003; Elliott et al. 2024). Historical evidence further shows that religious nursing sisters and women in faith‑based movements exercised significant leadership within alternative institutional spaces (Nelson 2003), challenging simple associations between leadership, masculinity and hierarchy. Importantly, hierarchical arrangements reflected broader sociopolitical conditions; military organisation amplified existing hierarchies within nursing rather than creating them outright.

While autocratic leadership in the context of the 1920–1950s ensured tight compliance and regimented control and order, today it is thought to constrain collaboration and participatory dialogue, frequently resulting in diminished morale, reduced job satisfaction and adverse effects on patient care and clinical outcomes (Al‐Thawabiya et al. 2023). Today, autocratic leadership in nursing often emerges under constraints such as limited delegation authority and inadequate training (Briker et al. 2021).

#### 4.1.2 Bureaucratic Leadership

The aftermath of the Second World War (1939–1945) brought the expansion of healthcare systems, marking an initial step toward the professionalisation of nursing and a rise in bureaucratic nursing leadership (Tobbell 2023). The concept of patient safety began to emerge, and nurse leaders were required to manage increasingly multifaceted organisational requirements (Swift et al. 2025).

Like autocracy, bureaucratic leadership prioritises structure and control, but places greater emphasis on hierarchical organisation (Khan et al. 2015). Bureaucratic leaders expect strict adherence to frameworks and standard operating procedures. This style is common in large organisations and healthcare settings, where compliance with rules and protocols is critical to preventing errors and ensuring successful outcomes (Khan et al. 2015). However, drawbacks include a tendency to prioritise steadiness over adaptability, resulting in inflexibility and reluctance to adopt new practices (Uhl‐Bien et al. 2020; Ominyi et al. 2025). For example, mandated use of clinical pathways may foster compliance and alignment with established protocols, a core tenet of bureaucratic leadership, however, it may also inhibit nurses' ability to adapt care delivery to specifically address individual patient needs. Barclay and Moore (2024) argue that bureaucratisation centralises power within an organisation's administrative structures, while Uhl‐Bien et al. (2020) contend that bureaucratic leadership styles restrict the autonomy of both nurses and nurse leaders. As such, bureaucratic leadership acted as a barrier to the professionalisation of nursing (Davis and Cushing 1999).

While there have been significant changes in nursing leadership styles over time, aspects of bureaucratic leadership, such as the fealty to strict policies and procedures, remain steadfast in healthcare, as a mechanism to protect both patients and staff. As a result of this, staff may feel disconnected, working in fragmented teams in isolation, and perceive that their voices are not heard (Barclay and Moore 2024).

These leadership approaches emerged in response to wartime exigencies and early hospital expansion, where rapid decision‐making, discipline and uniformity were prioritised over professional autonomy (Strahan 1997). Senior nurses exercising strict supervisory control over wards and training schools, with limited scope for discretion among junior staff, reflecting both military command structures and the subordinate positioning of nursing within medicine (Strahan 1997).

While bureaucratic leadership structures formalised nursing roles in the post‐war period, they also constrained professional autonomy. The emergence of transactional leadership in the 1960s–1970s can be read as both a response to growing system complexity and an attempt to reconcile demands for fairness and recognition with ongoing organisational control.

### 4.2 The 1960s and 70s—Social Change and Rebellion

#### 4.2.1 Transactional Leadership

Transactional leadership reflected the sociopolitical climate of the 1960s and 1970s, a period marked by civil rights movements, second‐wave feminism and countercultural shifts, challenging traditional hierarchies (Slonecker 2017). Healthcare systems were expanding rapidly, and nursing was transitioning from hospital‐based apprenticeship models to more formalised education. These changes created a tension between the need for operational stability and growing calls for professional autonomy and equity (Borbasi and Gaston 2002).

Characterised by a structured, task‐focused approach, transactional leadership manages performance through contingent rewards and sanctions (Dong 2023). Leaders clarify expectations, set short‐term goals and reward staff for meeting standards, while applying consequences for underperformance (Dong 2023). For example, a transactional nurse leader may offer rewards for timely completion of mandatory education and intervene only in instances of non‐compliance. This style is thought to promote order, accountability and operational efficiency, making it particularly attractive in regulated healthcare environments (Al‐Rjoub et al. 2024).

Rewards may range from praise and recognition to financial incentives or career advancement. In nursing, this approach is evident in appraisal systems, shift management and compliance monitoring, where clear expectations and structured roles are the norm (Häggström et al. 2025). A key strength of transactional leadership is providing clarity and stability in complex or high‐pressure situations. It ensures nurses understand their responsibilities, adhere to protocols and deliver consistent care. Additionally, it supports performance management systems like appraisals and shift scheduling, which are vital to operational efficiency (Richards 2020). When rewards are aligned with professional development, it can enhance job satisfaction, especially for staff who thrive under clear expectations and structured environments (Quinn 2023). Its adaptability across cultures also makes it valuable in diverse nursing teams (Anny and Kashif 2023). Transactional leadership also favours consistency and fairness (Richards 2020).

However, the limitations of transactional leadership are well documented. Emphasis on task completion and rule enforcement can lead to a rigid, non‐holistic approach to patient care which contributes to diminished staff empowerment, well‐being and job satisfaction, especially when penalties for underperformance are applied inconsistently or punitively (Richards 2020). This approach can be less effective if staff are not motivated by the rewards and incentives on offer (Anny and Kashif 2023). Transactional leadership can suppress creativity, collaboration and innovation due to its limited emphasis on emotional engagement and relationship‐building (Anny and Kashif 2023). Its emphasis on completing predetermined tasks may also restrict growth and reduce opportunities for exploring new ideas (Anny and Kashif 2023).

However, some purport this is not a limitation and transactional leaders can effectively lead organisational change (Dong 2023). For example, during the COVID‐19 pandemic, transactional leadership proved essential in enabling rapid decision‐making and maintaining control (Quinn 2023). This highlights that leadership styles can indeed be flexible and work in dynamic healthcare contexts and social eras.

Transactional leadership reflected a period of sociopolitical upheaval marked by civil rights movements, second‐wave feminism and challenges to traditional authority, while healthcare systems simultaneously sought organisational stability amid expansion and reform. This tension was evident in nursing using structured appraisal systems, formalised roles and performance‐based management, enabling some recognition of professional contribution while maintaining organisational control (Richards 2020).

### 4.3 The 1980s and 1990s—Transformation Leadership in a Changing World

#### 4.3.1 Transformational Leadership

Transformational leadership gained prominence during the late 20th century, a period marked by professionalisation of nursing and broader societal movements advocating for empowerment, equity and participatory governance (Rost 1993). The rise of university‐based nursing education in the 1980s and 1990s fostered autonomy and critical thinking, aligning with transformational ideals of inspiring innovation and shared vision (Bass 1985; Stievano et al. 2019). These changes reflected a cultural shift away from rigid hierarchies toward collaborative, values‐driven leadership, influenced by feminist movements and global calls for social justice (Gabel 2013).

Transformational leadership, described by Kok et al. (2023), encourages intellectual stimulation, challenging conventional practices and inspires innovation. Grounded in Bass's (1985) research, it emphasises role‐modelling and behaviours such as articulating a vision, building trust and mentoring staff. Four core processes define this style: idealised influence, inspirational motivation, intellectual stimulation and individualised consideration (Gabel 2013). Explained by Gabel (2013) and Kok et al. (2023), transformational leaders embody ethical principles, communicate vision effectively and support team growth through problem‐solving and personalised development.

While transformational leadership fosters purpose, commitment and excellence, it carries challenges. Emotional demands on nurse leaders can lead to burnout, and its reliance on idealised influence may suppress critical thinking (Cummings et al. 2018; Boamah 2022). These limitations highlight the need for context‐sensitive application. Despite this, transformational leadership remains associated with improved team performance and patient‐centred care (Bass 1985; Gabel 2013).

Transformational leadership rose alongside the professionalisation of nursing, the migration of education into universities and broader neoliberal reforms that emphasised innovation, accountability and professional identity. For example, nurse leaders advocated for degree‐based entry, supported nurses' engagement in research and policy work and promoted interdisciplinary collaboration, reframing nursing leadership as visionary rather than supervisory (Bass 1985; Gebbie 2009; Stievano et al. 2019). This professionalisation is captured in the below quote (Department of Community Services and Health 1990, 7).

The [Australian] Government accounted in 1984 that it would support the States in the transfer of nursing education from hospital based courses to courses in tertiary education institutions…it was envisaged that a more appropriately educated, flexible and carer orientated registered nurse would be produced…

These practices repositioned nurses as more autonomous professionals and knowledge contributors rather than task‑based workers (Gebbie 2009; Gabel 2013). Although often framed as progressive, transformational leadership's emphasis on vision, inspiration and idealised influence can obscure power asymmetries and place substantial emotional labour demands on nurse leaders (Boamah 2022). This raises questions about whose values are advanced within transformational agendas and whose voices remain marginal.

#### 4.3.2 Role‑Based Leadership

The 1980s marked a turning point for nursing and healthcare regulation (Stanley et al. 2022). Professional bodies and regulatory authorities strengthened their influence, introducing more rigorous standards for practice, competency frameworks and ethical codes. These changes were driven by the need for public trust, patient safety and professional accountability (Stievano et al. 2019). Accreditation requirements further reinforced these expectations, compelling organisations to align leadership structures with compliance mandates (Stievano et al. 2019; American Accreditation Association 2025). As a result, new leadership styles emerged, and existing ones were refined to meet evolving standards and accreditation benchmarks (Stievano et al. 2019; American Accreditation Association 2025). The importance of health leadership demonstrated in the below quote from the World Health Organisation (1988, p.3).

‘Leadership development for Health for all’ This is the title of the technical discussions taking place during the 41st World Health Assembly in Geneva in May. Each year, delegates to the Assembly, as well as representatives from other fields outside WHO, meet to discuss a key issue of political health. The theme of ‘leadership’ reflects the need to create a critical mass of people in each country who can inspire and motivate others towards the goal of Health for all.

Role‐based leadership is grounded in the principle that authority, accountability and decision‐making, and are tied to specific organisational roles rather than individual characteristics (Sheard and Kakabadse 2007). This approach emphasises the expectations, responsibilities and scope of authority inherent in each position (e.g., Nurse Unit Manager or Director of Nursing), ensuring clarity and consistency in leadership functions (Sheard and Kakabadse 2007). In healthcare, where patient safety and quality outcomes depend on seamless collaboration, the interdisciplinary nature of care delivery makes clearly defined roles and scopes of practice necessary. Each discipline, such as nursing, medicine and allied health, must operate within established boundaries to maintain accountability and prevent role ambiguity (Sheard and Kakabadse 2007). However, this style is reminiscent of early, hierarchical models of leadership, and may not fully appreciate the everyday leadership demonstrated by those not in a specific leadership role.

### 4.4 The 2000s—Turn of the Millennium

#### 4.4.1 Participative Leadership

Early in the 2000s, the worldwide nursing shortage was predicted, and the impacts of this began to be identified (Fowler et al. 2006). Maintaining an adequately qualified nursing workforce was a theme of concern across most first‐world countries, prompting urgent strategies to meet the growing operational demands of healthcare systems (Forrester and Griffiths 2001).

At the turn of the century, many nations reviewed how nursing would move into the new millennium, including leadership, governance and redesigned models of care (Fowler et al. 2006). This new era of healthcare identified the need for stronger yet flexible governance, the need for adapted models of care, and as such, the need for fresh forms of leadership to engage shared decision‐making and promote clinical effectiveness (Scott and Caress 2005). During this time, participative leadership emerged in nursing management, characterised by shared decision‐making between managers, leaders and employees, delegation of some decision‐making authority to front‐line nurses in practice and encouraging active employee involvement in collaborative decision‐making (Kahai et al. 1997). Borbasi and Gaston (2002) purported that two core characteristics underpin participative leadership: consultation with staff to problem‐solve as a team prior to decision‐making, and adequately resourcing and supporting teams to actively undertake the work decided.

Participative leadership style fosters trust and a sense of value within the nursing team, resolving problems through democratic consultation, with ultimate decision‐making being the responsibility of the nurse leader (Borbasi and Gaston 2002). Participative leadership is, however, described as effort‐intensive. Successful implementation requires constant encouragement and support to engage staff and ensure the success of the model (Huang et al. 2010). The benefits however are described as ownership, employees feeling valued and accountable when working toward and meeting goals, and a collaborative approach to problem‐solving which builds capable teams. Studies have reported participative leadership is positively associated with employee creativity, empowerment and mental well‐being (Miao et al. 2014; Usman et al. 2021), improving performance and innovation at an organisational level (Yan 2011; Miao et al. 2014).

As the complexities and barriers to providing safe and effective healthcare deepened in the 2000s, participative leadership was considered an effective approach for improving management decisions (Huang et al. 2010), where some control leadership becomes the collective work of the team.

#### 4.4.2 Laissez‐Faire Leadership

On the greater end of the spectrum, laissez‐faire leadership emergent, reprinting a decidedly hand‐off approach to leadership (Alsadaan 2025). Grounded in the belief that individuals perform best with minimal oversight, this style is thought to promote autonomy and innovation, proving effective in the right contexts and with capable teams (Zheng and Li 2024). However, its hands‐off nature is often criticised for lacking clear direction and accountability (Zheng and Li 2024).

First introduced by psychologist Kurt Lewin in 1939, laissez‐faire leadership involves delegating decision‐making and limiting direct intervention (Bass and Avolio 1990). The term, derived from the French ‘let it be’, describes a leader who minimises involvement in team operations (Ndlovu 2024). Success with this style depends heavily on team members' competence, motivation and ability to self‐manage. Laissez‐faire leadership emerged in the 2000s, as healthcare systems embraced shared governance and decentralised decision‐making, with the intent of empowering nurses with autonomy and professional judgement (Anthony 2004; Zheng and Li 2024). Technological advancements and the rise of highly skilled, self‐managing teams enabled leaders to adopt such an approach, while still maintaining accountability and supporting staff innovation (Anthony 2004). This resurgence was not a lapse into inaction, but a deliberate shift toward granting experienced nurses' greater autonomy within structured boundaries (Alsadaan 2025). Several social and cultural factors contributed to this trend: the maturation of nursing education and professional standards, which equipped frontline nurses with confidence and competence to make independent decisions; a growing desire among nurse managers to empower rather than micromanage, fostering psychological freedom and trust, and broader societal movements emphasising collaboration and professional agency (Mrayyan et al. 2024). Contemporary discourse reframed laissez‐faire leadership from ‘absence of leadership’ to a form of strategic detachment, where guidance and boundaries remain clear, but nurses exercise autonomy in clinical judgement (Alsadaan 2025).

When implemented effectively, laissez‐faire leadership can enhance innovation, professional growth and job satisfaction. It encourages initiative, accelerates decision‐making and fosters a culture of trust and accountability, particularly valuable in agile or remote settings (Jamali et al. 2022). Employees who feel trusted and empowered are typically more motivated and engaged, contributing to a respectful and productive workplace (Yang 2015). Additionally, reduced oversight can streamline operations and promote self‐management, provided employees possess the necessary skills and knowledge (Jamali et al. 2022). Conversely, poor implementation can lead to confusion, lack of direction and diminished performance, especially among inexperienced or disengaged teams (Khan and Tidman 2021; Musinguzi et al. 2018). A lack of accountability may result in overlooked tasks and duplicated efforts. Leaders perceived as detached may harm morale and cohesion, further undermining team effectiveness.

Today, laissez‐faire is described as outdated and ineffective (Zhang et al. 2023), with recent research revealing its dual nature, both empowering and potentially destructive (Zheng and Li 2024; Sun et al. 2025), highlighting the divergent perspectives of its impact (Zhang et al. 2023; Sun et al. 2025).

### 4.5 The 2010s—The Leader as Servant

#### 4.5.1 Servant Leadership

**Figure 1:**
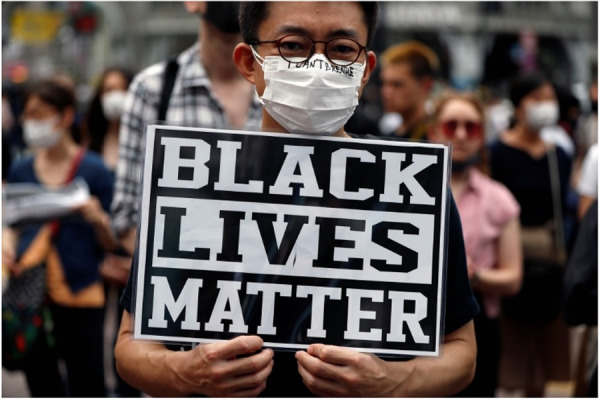
#blacklivesmatter. From *Updated: Australia, Asia Protests Embrace ‘Black Lives Matter’ Movement*, by Kelly and Saito (2020).

The 2010s signalled an interesting new phase for nursing leadership. Deeper developments into person‐centred nursing theories, in particular McCormack and McCance (2010), acted to further embed models which centred care on the needs of individuals and families. At this time, heightened awareness and advocacy for social disparities of marginalised people saw healthcare move further into equity‐based models; as examples, the rights, needs and wants of communities such as LGBTQIA+, First Nations peoples and refugees were highlighted. Indeed, social movements such as #metoo and #BlackLivesMatter (see Figure 1) brought the often hidden and unjust harms of society to the forefront, sparking debate, reflection and deeper engagement in the social aspects of health and healthcare (Khubchandani et al. 2019; Gatwiri et al. 2021).

These global movements impacted upon healthcare systems, including nursing, particularly around issues of equity, justice and inclusion. Social responsibility has always been at the core of the nursing profession (Kim 2023), and as such, social movements around structural inequalities and implicit biases shape patient care and workforce dynamics, prompting nursing leaders to re‐evaluate traditional leadership models. The profession began to more actively engage with the social determinants of health, particularly cultural competence (Dauvrin and Lorant 2015; Curtis et al. 2019), recognising leadership must extend beyond clinical competence to encompass advocacy, cultural safety and ethical stewardship.

In addition to the social movements, the stressors of nursing came under scrutiny, with burnout contributing to nurses leaving the profession, widening the nursing shortage gap (Bentley 2010; Guo et al. 2018). The notion of resilience was highlighted (Turner 2014, 71), with the intent of strengthening a nurse's ability to ‘bounce back’ after adversities. Research into how nurses become resilient, and maintain resilience overtime became popular, with scholars increasingly recognising resilience as a dynamic, context‐dependent process rather than a fixed trait (Hart et al. 2014; Manomenidis et al. 2019). Leadership in this space and time thus moved into more social, supportive structures, with servant leadership becoming a dominant discourse. The importance of leadership in alleviating burnout in nurses is identified by Hart et al. (2014, 721).

Despite economic hardships, challenges in the workplace and modern issues, nurses continue to serve the public and to do extraordinary work with few resources, but the stress of the job creates challenges for the retention of nurses. If experienced nurses have difficulty coping with todays pressures, new graduates are even more at risk for burnout.

Aligning with moral philosophies at the turn of the 20th century, servant leadership was initially described by Greenleaf in their 1970 essay ‘The Servant as Leader’ which focused on the development and ethical growth of followers (Boyum 2008). Greenleaf concluded that the essence of leadership lies in the desire to serve first and that genuine leadership emerges from this orientation (Boyum 2008). In more modern scholarship, Spears (2004) and later, van Dierendonck (2011), synthesised servant leadership research, identifying the key traits as empowering and developing people, humility, authenticity, interpersonal acceptance, providing direction and stewardship. These traits distinguish servant leadership from other models like transformational or transactional leadership, which often prioritise organisational goals over individual well‐being. Servant leadership aims to move away from management style toward more relational and ethical approaches to leadership. It prioritises listening, empathy, stewardship and commitment to the personal and professional development of staff and patients alike (Canavesi and Minelli 2022).

Servant leadership gained traction in the nursing literature from 2012 through the work of Sherman (2012), and in 2017, where nursing scholars asked, ‘whom do we serve?’ (Savel and Munro 2017, para. 6). This provided a natural alignment for the nursing profession, which is traditionally built on servitude and service to patients and the wider medical models. However, servant leadership was not altogether altruistic, with research focusing on the positive impact of servant leadership for nurses and quality and safety outcomes. For example, Du et al. (2024) found that servant leadership may enhance nurses' compliance with standard precautions in daily practice. Similarly, Todt and Covington (2023) argue for the integration of servant leadership into nursing education, suggesting it cultivates ethical, reflective practitioners equipped to lead in complex healthcare environments.

Servant leadership also supports collaborative and inclusive team cultures, essential in multidisciplinary healthcare settings. It encourages leaders to act as facilitators rather than directors, promoting shared decision‐making and mutual respect (van Dierendonck 2011). However, despite growing popularity, servant leadership remains under‐theorised in empirical research. Scholars continue to explore its philosophical foundations, practical applications and outcomes in diverse organisational contexts (Canavesi and Minelli 2022). In summary, the 2010s ushered in equity‐focused care and resilience discourse, with servant leadership emerging as a dominant model that prioritised empathy, ethical stewardship and the personal development of staff and patients.

#### 4.5.2 Authentic Leadership

Authentic leadership emerged from psychological and philosophical origins, but its formal study began in the early 2000s, notably through Luthans and Avolio (2003), which introduced a framework incorporating self‐awareness, balanced information processing, relational transparency and moral integrity to meet the demands of complex 21st‐century organisations. This development reflected broader societal calls for ethical and transparent leadership following corporate scandals. By the 2010s, authentic leadership extended strongly into nursing, where it was embraced to cultivate trust, resilience and positive organisational cultures in healthcare environments (Almutairi et al. 2025). Defined as a leadership style grounded in self‐awareness, transparency, ethics and balanced decision‐making, authentic leadership emphasises being true to oneself while fostering trust and genuine relationships with followers (Gardner et al. 2011). Authentic leaders are guided by internal moral values and strive to create a positive and ethical organisational climate, demonstrating honesty and reflecting personal values such as transparency, self‐awareness, internalised moral perspective and care (Gardner et al. 2011; Leroy et al. 2015; Almutairi et al. 2025).

There is a presenting argument with authenticity and leadership. Leaders often seek to align followers' values with their own in pursuit of a shared vision and organisational objectives; however, this process can inadvertently increase pressure on followers and leaders to compromise their personal authenticity (Gardner et al. 2021). Due to competing values and goals, a power imbalance may occur. However, within authentic leadership relationships, respect for the leader and follower occurs, and the leader will usually seek to reconcile (Deci and Ryan 2000). This leadership style is grounded in self‐determination theory (Deci and Ryan 2000), as humans achieve authenticity when the need for autonomy, competence and relatedness is fulfilled. In addition, this also includes person‐centred theory as this leadership style is holistic and includes multiple interactions within the teams (Meyer and Morin 2016).

The rise of servant and authentic leadership coincided with growing attention to social justice, cultural safety, workforce well‑being and increasing recognition of burnout in nursing. Leaders responded by prioritising staff well‑being, fostering psychologically safe environments and engaging in equity‑focused advocacy, embedding leadership in relational and ethical commitments rather than authority alone. Servant and Authentic leadership are similar in that they both focus on harmonising leader–follower relationships and are fundamentally people‐centred. Servant leadership has service as its foundation, while authentic leadership premises self‐awareness and authenticity as the core drivers of leadership. Authentic leadership emphasises self‐awareness, transparency and ethical decision‐making, fostering trust and genuine relationships while promoting autonomy and holistic team engagement. While servant and authentic leadership emphasise ethics, relationality and self‐awareness, such approaches may also risk individualising responsibility for well‐being within structurally constrained systems. Without parallel attention to organisational and policy conditions, these models may inadvertently place the burden of moral and emotional labour on individual leaders. Servant and authentic leadership were enacted through practices such as open‐door leadership, reflective supervision, shared governance forums and explicit prioritisation of staff well‐being and psychological safety. Leaders increasingly engaged in advocacy related to equity, inclusion and burnout, aligning everyday leadership actions with broader person‐centred and social justice priorities.

### 4.6 The 2020s—Age of Change

#### 4.6.1 Adaptive and Emotionally Intelligent Leadership

The 21st century is characterised by unprecedented speed and complexity; This relentless pace is compounded by digital saturation, ambiguous purpose, organisational turbulence and generative artificial intelligence. These conditions, coupled with the unprecedented landscape of the COVID‐19 necessitated the need for adaptive, innovative leadership approaches to navigate uncertainty and sustain effective healthcare delivery (Worman and Sturmberg 2025). Contemporary leadership approaches have been shaped by intersecting sociopolitical pressures, including global health crises, workforce shortages, accelerated digitalisation and political uncertainty. These conditions required nurse leaders to rapidly redeploy staff, implement flexible rostering models and balance directive safety measures with empathetic support, illustrating adaptive leadership enacted under acute systemic strain. The response in an always‐changing environment is described by Worman and Sturmberg (2025, 3).

In uncertain and chaotic situations, variability or diversity of response, is an essential part of the trial‐and‐error activity which leads to new adaptive patterns of behaviour. As hierarchical structures are created, they have the power to establish mandates aimed at creating conformity.

Adaptive leadership is said to mobilise people to tackle complex challenges and thrive amid change. This style emphasises flexibility, learning and collaboration, rather than relying solely on authority or technical expertise (Seibel et al. 2023; Worman and Sturmberg 2025). Adaptive leadership theory posits that everyone has a problem‐solving style, each person generates different ideas to solve a problem (Kirton 1976) and emphasises the leader's role in guiding adaptation rather than focusing solely on internal and external motivators (Seibel et al. 2023). Healthcare systems exist in an ever‐changing environment where policy shifts, hierarchical adjustments and resource constraints necessitate adaptive leadership (Worman and Sturmberg 2025).

According to the literature, adaptive leaders acknowledge uncertainty but seek solutions, while promoting resilience in staff (Huston and Sherwood 2025). This became an important issue within the profession, as more nurses began to experience burnout due to rapid changes (Yuguero et al. 2022). Adaptive leaders must also, however, adapt themselves, and this is not a simple task for some (Huston and Sherwood 2025). Change and adaption takes time, commitment, emotional intelligence flexibility and collaborative approaches to help nurse leaders navigate rapid change.

Within this, emotionally intelligent leadership highlights the importance of internal regulation in performing leadership duties. It emphasises understanding and managing emotions, both your own and those of others, to create positive, productive and collaborative environments and includes self‐awareness, self‐management, social awareness and relationship management (Cummings et al. 2005). The connection between emotional intelligence and resilience is made. Resilient leaders leverage emotional intelligence and empathy to understand what individuals and teams may feel during difficult times, fostering trust and cohesion through supportive response to staff concerns (Cummings et al. 2005). Emotionally intelligent leaders are attuned to how teams are feeling and apply both resonant and dissonant styles of emotional intelligence as needed to provide a greater level of support (Blizzard and Woods 2020). Resonant styles such as visionary, coaching, affiliative and democratic promote emotional connection, whilst dissonant styles including pacesetting and commanding do the opposite (Blizzard and Woods 2020). Resonant leadership is strongly associated with emotional intelligence, a key component of empathy (Blizzard and Woods 2020).

#### 4.6.2 Ambidextrous Leadership

Ambidextrous leadership, first introduced by Duncan (1976), who purported its use for innovation, is underpinned by Ambidextrous leadership theory (Alghamdi 2018). Ambidexterity, defined as having the ability to use both hands with equal ease (Rosing et al. 2011), in leadership refers to a leader's ability to balance two seemingly opposing behaviours, exploration and exploitation, within an organisation (Alghamdi 2018). Ambidextrous leaders demonstrate flexibility and can alternate between leading a team and providing individualised support (Rosing et al. 2011).

**Figure 2:**
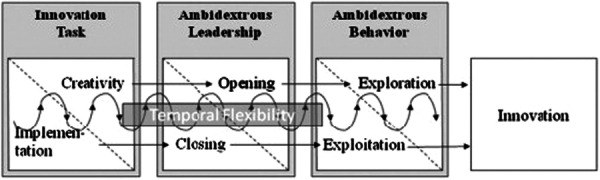
Ambidextrous model. From ‘Explaining the Heterogeneity of the Leadership‐Innovation Relationship: Ambidextrous Leadership’, by Rosing et al. (2011).

Ambidextrous leadership is essential for fostering innovation, involving three core elements: (1) adoption of opening leader behaviours to encourage exploration, (2) application of closing leader behaviours to ensure effective exploitation and (3) temporal flexibility to alternate between these orientations (see Figure 2) (Rosing et al. 2011). Such opening and closing behaviours are not necessarily opposed, but rather a reflection of the needs of any given situation. For example, through opening behaviour a leader may encourage diverse approaches to tasks, inspire calculated risk‐taking and encourage independent thought, while closing behaviours may include establishing standardised routines, implementing corrective measures and sanctioning errors (Rosing et al. 2011; Alghamdi 2018).

Ambidextrous leaders engage in complex thinking processes to support team diverse needs and goals. The adaptation of leadership styles requires understanding that at the employee level, employees can learn a new skill or knowledge as well as adapt these to the present routine. They can also learn on the job, generate and implement new ideas (Alghamdi 2018).

During periods of rapid disruption, including the COVID‐19 pandemic, adaptive and ambidextrous leadership were enacted through rapid workforce redeployment, flexible rostering, decentralised decision‐making and the use of digital communication platforms (Worman and Sturmberg 2025). Nurse leaders balanced directive leadership required for safety with supportive practices that attended to staff uncertainty, fatigue and moral distress (Shanafelt et al. 2020). Today, such styles reflect the ability to oscillate between innovation and standardisation, enabling leaders to foster creativity while maintaining patient safety and operational efficiency. Both styles now prioritise flexibility, ethical decision‐making and contextual responsiveness, aligning with contemporary demands for agility, inclusivity and evidence‐based practice in nursing leadership.

## 5 Discussion

Rather than presenting nursing leadership as following an evolutionary or progressive trajectory, this article examined approaches as responses to specific historical, sociopolitical and institutional conditions, rather than as stages in a linear progression toward ‘better’ leadership. From this perspective, leadership change is understood as adaptive and contextual rather than accumulative or value ranked.

This paper set out to critically examine the evolution of nursing leadership styles over the past century, aiming to understand how historical, social and professional contexts have shaped nursing leadership. In doing so, we highlighted how nursing leadership transitioned from rigid, hierarchical models in the early 20th century, to more relational and adaptive approaches in recent years. Each era reflected the prevailing sociocultural norms and healthcare priorities, from wartime efficiency and hierarchical control, through professionalisation and transformational ideals, to inclusivity, resilience and innovation. Indeed, leadership styles and theories are reflections of the time, acting to represent the prevailing social movements, issues and requirements of any given era. Nursing leadership has adapted and changed with these times, moving with the rhythm of the world around it. Simultaneously, the mapping demonstrates that the history of the profession is often carried forward in subsequent leadership theories and practices. The deep institutional roots of healthcare hierarchy remain, and while nursing as a profession grows, the past often informs us who we are today (Vivier et al. 2024). Nurses are often positioned within a tension of historical oppression (which acts to discount and subdue the importance of nursing work) and at the cusp of innovation and advancement (which acknowledges the potential of nurses to disrupt and improve entrenched practices and structures) (Gebbie 2009; Leary et al. 2024). As such, modern leadership can embody many forms and styles, considering the personal attributes and education of the leader, the clinical context and the structure and system within which we work (Singh et al. 2023).

Leadership in nursing is shaped by multiple interrelated factors that influence both practice and professional identity. Organisational culture and policy establish the framework within which leadership operates, impacting job satisfaction, retention and care quality (Aydogdu 2023). Workforce dynamics and interprofessional relationships further determine collaboration and role clarity, with nurse leaders playing a critical role in fostering high‐reliability teams and safe care environments (Joseph et al. 2022). Leadership approaches may differ between contexts, for example acute and community settings, requiring context‐specific strategies to address staffing, resource allocation and patient needs (Guibert‐Lacasa and Vázquez‐Calatayud 2022; American Organization for Nursing Leadership 2015).

The complexity of healthcare necessitates the need for flexibility and adaptability, where intuitive leaders know when to control, direct and step back. Rather than viewing leadership as a fixed point in time, our analysis highlights that leadership exists on a spectrum, it is dynamic, context‐dependent and deeply relational. Indeed, leadership styles are not mutually exclusive. Effective nurse leaders often blend elements of transactional clarity, transformational vision and authentic engagement to meet the demands of healthcare environments. This fluidity reflects the reality that leadership is shaped by the interplay of organisational culture, workforce expectations and external pressures, rather than by rigid adherence to a single model. On any given day, a nurse leader may flip between authoritarian, transactional and laissez‐faire leadership styles, recognising situations and followers require different skills and directions from a leader.

Flexibility in the modern age is essential. Global crises, rapid digital transformation and persistent workforce shortages have created volatile, uncertain, high‐stakes environments where traditional leadership paradigms alone may be insufficient (Al Mazrouei 2023; Khalil et al. 2026). Contemporary leadership styles, such as emotionally intelligent and ambidextrous leadership, have emerged to address these challenges by prioritising resilience, empathy and innovation while maintaining patient safety and operational efficiency. These approaches recognise leadership is not merely about authority or motivation, but about navigating systemic complexity, fostering psychological safety and enabling teams to thrive amid uncertainty. So too, leadership theories continue to change, leveraging the old and new. A revival of stoic theories encourages nurses to focus on the dichotomy of control, acting with reason to maintain calm communication and decision‐making (Frisina 2024). Popularised theories such as *Let Them* (Robbins and Robbins 2024) have emerged from the post‐COVID chaos, encouraging nurses to focus inward, set boundaries and reduce stress—for example, *Caroline Nurse Coach* Blog, 2025 and Podcasts such as *A Nurses Lift to Work*, Danna (2025).

On the other hand, nursing is faced with everyday opportunities where leadership, advocacy and authority are required. Recently, the US administration endorsed the removal of nursing from the list of professional degrees, citing federal loan cap purposes (U.S. Department of Education 2025). Opposition to this has been swift and strong, with international leaders affirming nursing as a value‐driven profession that plays an essential role in the healthcare system. Indeed, the reclassification negates the ‘depth of nursing's disciplinary knowledge and its role in promotion health, safety and equity’ (Council of Deans of Nursing and Midwifery Australia and New Zealand 2025, para. 2). The declassification is reminiscent of the historical roots of nursing, where subordination, compliance and order were prioritised ‐ a positioning which does not fit within the modern world of healthcare. Similarly, international nurse leaders and professional organisations have played visible roles in advocating for workforce protections, safe staffing ratios and professional recognition in the wake of the COVID‐19 pandemic, positioning leadership as both politically engaged and socially responsive rather than purely managerial. While these examples are at the macro level, nursing leadership occurs across all levels and often necessitates different approaches and styles. The same is certainly true for daily decision‐making in hospital wards, community health and aged care (to name a few). Leadership as such must be agile, responsive and purposeful.

A critical reading shows that no leadership style is value‑neutral with each carrying assumptions about power and agency that shape who is heard, who leads and how care is organised. The persistence of hierarchical norms, even within relational or people‑centred models, suggests that shifts in leadership language do not necessarily shift underlying power structures. This highlights the need to examine not only which styles are adopted but how they operate within existing institutional constraints.

A key limitation of historical analysis is the risk of over‑attributing leadership developments to singular sociopolitical events when leadership discourses emerge from intersecting influences. While this paper highlights major markers such as war, social movements and professional reform, these are used as heuristic anchors rather than exhaustive or causal explanations. Leadership styles are interpreted as contingent responses shaped by multiple, co‑occurring forces, not direct outcomes of any one event. This approach aligns with scholarship that views professional leadership development as negotiated, layered and institutionally mediated rather than linear or monocausal.

The analysis has implications for education, practice and leadership development. Seeing leadership as historically contingent supports curricula that emphasise adaptive, reflective capability. For nurse leaders, recognising enduring hierarchical legacies can encourage more intentional relational and context‑responsive approaches. At a systems level, treating leadership as dynamic rather than prescriptive can enable more flexible governance suited to workforce complexity. Understanding leadership evolution as non‑linear invites scrutiny of how nursing responds to sociopolitical change. Historically, leadership shifts often occurred cautiously, with delays between social movements and their formal uptake in leadership models. These lags raise questions about whose values shape leadership discourse and how professional structures both enable and constrain responsiveness to cultural change.

This historical trajectory reflects dominant discourses within Western healthcare contexts. Leadership developments have varied across countries, shaped by local political, cultural and institutional conditions. The analysis is therefore situated within Western systems and offers a broad interpretive synthesis rather than a universal or global pathway. Taken together, this history suggests not a linear progression toward more enlightened leadership models but a pattern of adaptive responses to shifting sociopolitical and professional demands. Leadership approaches emerged at particular moments because they addressed tensions, between control and autonomy, care and efficiency, equity and hierarchy, embedded in healthcare systems. From this perspective, leadership change reflects ongoing negotiations of power, identity and legitimacy within nursing rather than the accumulation of increasingly superior techniques.

## 6 Critical Reflection

As authors, our analysis is shaped by our professional and academic positioning within nursing education, leadership and practice. Our interpretations are informed by experience working within contemporary healthcare systems that continue to reflect many of the historical hierarchies and leadership discourses examined in this paper. Acknowledging this positionality is essential, as it influences both how historical leadership models are interpreted and how their relevance to contemporary practice is understood.

The historical analysis presented in this article offers a lens through which to examine the evolution of nursing leadership, but its true value lies in how it might prompt reflection on contemporary practice. We encourage readers to reflect on their own leadership experiences, the environments in which they operate and the histories and contexts to which they belong. We invite readers to consider the following questions:

1. What does leadership mean to you?
2. How do the leadership styles outlined—from autocratic to adaptive—manifest in your workplace?
  a. Which leadership styles dominate, which are absent and what assumptions about power, authority and care underpin these patterns?
  b. Reflect on whether these approaches align with the needs of your team, those you care for and the broader health system.
3. Which leadership styles and theories do you gravitate toward? Why?
4. Consider the spectrum of leadership rather than fixed categories.
  a. Do you find yourself blending styles, moving from transactional clarity during high‐pressure situations, to authentic or emotionally intelligent approaches when supporting staff well‐being?
  b. How does your organisational culture enable or constrain this flexibility?
5. What are the emerging challenges for your area, organisation, profession? What will leadership look like for these challenges?

## 7 Limitations

A limitation of this historical analysis relates to the availability and accessibility of early archival primary sources. While nursing leadership practices from the early 20th century were informed through secondary historical accounts, the oldest archival primary source directly analysed in this review dates from 1976. Earlier nursing leadership materials were often unpublished, locally archived or embedded within broader institutional or medical records, limiting their accessibility. Consequently, interpretations of leadership practices prior to this period necessarily rely more heavily on secondary historical analyses.

As this study does not involve original archival or oral history research, its interpretations are necessarily shaped by the availability and framing of published historical sources. Because this analysis does not draw on extensive archival records of individual nurse leaders, it cannot determine how specific leaders enacted leadership in daily practice. Instead, it offers a historically informed interpretation of leadership language, structures and professional narratives reflected in published sources. Individual leaders may have acted in ways that diverged significantly from dominant discourse.

Leadership styles are described as ‘prominent’ or ‘dominant’ based on their visibility and recurrence in nursing leadership scholarship, professional publications, policy documents and leadership theory of the period. Dominance refers to patterns in discourse, not uniform enactment across leaders, settings or countries. This approach traces discursive prominence rather than attempting to verify individual leadership behaviours, aligning with historical methods focused on interpreting how leadership was articulated and theorised within its time.

The greater number of leadership styles identified after 2000 does not imply that earlier leadership was less complex. Instead, it reflects increased conceptual differentiation in contemporary scholarship, rapid sociotechnical change and growing attention to naming and distinguishing leadership approaches. Earlier discourse tended to use broader, less differentiated constructs, whereas recent decades have produced more granular typologies in response to rising complexity, workforce pressures and organisational reform.

## 8 Conclusion

Leadership in nursing is a dynamic and multifaceted construct, subject to the history, social and cultural influences of the nursing profession and broader healthcare sector. Our analysis demonstrates that leadership styles have evolved from rigid, hierarchical models, to more relational, adaptive approaches. Each era reflects the prevailing norms and priorities of its time, yet the deep institutional roots of healthcare hierarchy continue to influence leadership practice today. Contemporary challenges, including global health crises, digital transformation and workforce shortages, demand leadership that is flexible, evidence‐informed and contextually responsive. We argue that while contemporary models may focus on emotional intelligence, adaptivity and ambidexterity, leadership styles may be context‐ and situation‐specific and are often signals of broader movements in the healthcare sphere. Ultimately, nursing leadership is not merely a positional function but a relational, values‐driven process. By making visible the historical roots of contemporary leadership practices, this paper provides nurse leaders, educators and policymakers with a framework for critically examining not only how leadership is enacted, but why certain approaches persist. This historical analysis encourages readers to reflect on leadership in nursing and consider how internal and external factors may impact upon one's leadership style and choice. As we look ahead, the challenge for nurse leaders is to balance adaptability with authenticity, blending lessons from the past with innovations for the future.

## Funding

The authors have nothing to report.

## Ethics Statement

Ethical approval was not required for this study because it involved analysis of publicly available historical and scholarly sources and did not include human participants, patient data or identifiable personal information. The study was conducted in accordance with accepted principles of research integrity, scholarly rigour and responsible historical inquiry.

## Conflicts of Interest

The authors declare no conflicts of interest.

## Generative AI Disclosure

The authors declare that the generative artificial intelligence (AI) tool, CoPilot, was used in this manuscript for paragraph structure only. All content has been critically reviewed, revised and verified by the authors.

## Data Availability

The data that support the findings of this study are available from the corresponding author upon reasonable request.
